# Neurons in the Amygdala with Response-Selectivity for Anxiety in Two Ethologically Based Tests

**DOI:** 10.1371/journal.pone.0018739

**Published:** 2011-04-11

**Authors:** Dong V. Wang, Fang Wang, Jun Liu, Lu Zhang, Zhiru Wang, Longnian Lin

**Affiliations:** Key Laboratory of Brain Functional Genomics (Ministry of Education), Institute of Brain Functional Genomics, East China Normal University, Shanghai, China; Pontifical Catholic University of Rio Grande, Brazil

## Abstract

The amygdala is a key area in the brain for detecting potential threats or dangers, and further mediating anxiety. However, the neuronal mechanisms of anxiety in the amygdala have not been well characterized. Here we report that in freely-behaving mice, a group of neurons in the basolateral amygdala (BLA) fires tonically under anxiety conditions in both open-field and elevated plus-maze tests. The firing patterns of these neurons displayed a characteristic slow onset and progressively increased firing rates. Specifically, these firing patterns were correlated to a gradual development of anxiety-like behaviors in the open-field test. Moreover, these neurons could be activated by any impoverished environment similar to an open-field; and introduction of both comfortable and uncomfortable stimuli temporarily suppressed the activity of these BLA neurons. Importantly, the excitability of these BLA neurons correlated well with levels of anxiety. These results demonstrate that this type of BLA neuron is likely to represent anxiety and/or emotional values of anxiety elicited by anxiogenic environmental stressors.

## Introduction

Anxiety is a normal emotional response to potential threats or stresses, and is associated with a variety of psychological disorders when it becomes excessive [1], [2]. The amygdala plays an essential role in mediating emotions such as anxiety [3]–[7]. Recently, basolateral amygdala (BLA) has been widely studied as one of the critical components in the neural circuitry medicating anxiety-related states and behaviors [8]–[10]. Lesion and pharmacological studies indicated that BLA activation increases, whereas its inhibition decreases, anxiety-related physiological and behavioral responses [11]–[13]. However, the neural mechanisms that mediate anxiety states and behaviors remain unclear in the BLA.

Laboratory rodents are commonly used to study anxiety-related behaviors[14], [15]. Anxieties in these animal models can be classified as either conditioned or unconditioned responses to stimuli [16]. Ethologically based models of anxiety (unconditioned) are usually associated with nonspecific events or stimuli (e.g., open-field test) [16]. An animal's natural reactions to these non-specific stimuli may affect various behavioral indexes (e.g., thigmotaxis, defecation) that are commonly used to estimate anxiety [14], [16]. It has been reported that prolonged exposure (15 min) of rats to an open field caused anxiety and increased immediate-early gene (e.g., c-Fos) expression in the anterior part of the BLA [17]. However, electrophysiological activity of individual neurons in the BLA has not been examined in freely-behaving animals under ethologically based conditions of anxiety.

To investigate the potential role of amygdala neurons in anxiety, we carried out multi-channel *in vivo* extracellular recordings in the BLA of freely behaving mice subjected to two ethologically-based tests of anxiety. We found that one distinct group of BLA neurons displayed a characteristic slow-onset and progressively- increased firing rate, which was directly correlated to a gradual development of anxiety-like behaviors in multiple tests of anxiety such as open-field and elevated plus-maze tests.

## Results

### Ethological measures of anxiety in the open field and enriched box

**Figure 1:**
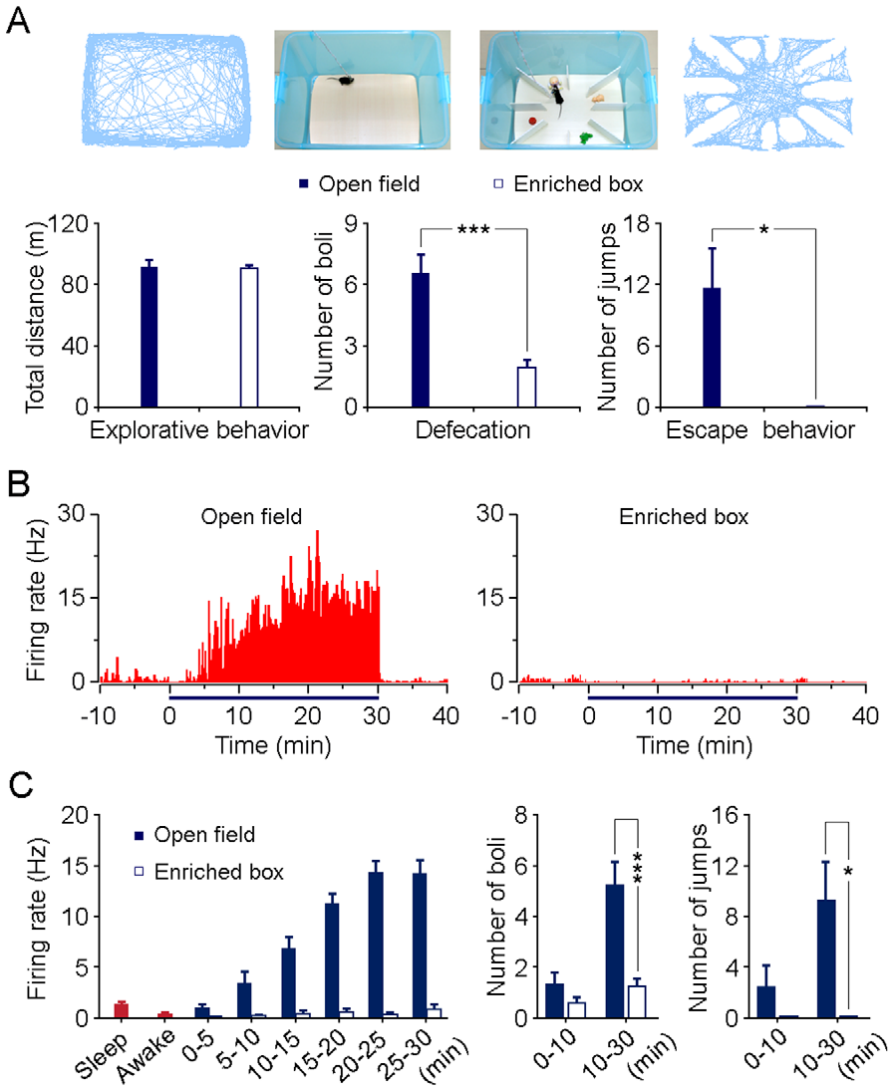
Activity of type-1 BLA neurons correlates with anxiety-like behaviors in the open-field test. (A) Upper panels, typical travel traces in an open field (left) and an enriched box (right) where the mouse was exposed for 30 min. Lower panels, statistical analyses of locomotor activity, defecation and escape behaviors in the two environments (n = 12; mean ± SEM). *P<0.05, ***P<0.001 by *t*-test. (B) A type-1 BLA neuron shows a tonic firing with a gradual increase in firing rate in an open field (left), but no activation in an enriched box (right). Horizontal lines below the histograms indicate the exposure duration (30 min). (C) The gradual increase in firing rate of type-1 BLA neurons (left panel; n = 10) was paralleled by a gradual build-up of anxiety-like behaviors in the open field (right panels; n = 12). Data in all panels are plotted as mean ± SEM (error bars). *P<0.05, ***P<0.001 by *t*-test.

The open-field test, originally designed by Hall on rats [18], is a widely used animal model to study ethologically-based anxiety [19], [20]. Typically, in an open field, the total exploratory travel distance is considered to be locomotor-related, whereas the levels of defecation, escape behaviors and thigmotaxis (the tendency to stay on the periphery of the open field; Figure 1A, upper-left) are considered to be anxiety-related [21]–[23]. In order to exclude the impact of locomotor activity on anxiety behaviors, we have designed an enriched box as a cross reference. The enriched box, with the same size as the open field box, was divided into eight small rooms and enriched with toys (Figure 1A, upper-right). Our results revealed that the total distances traveled by the mice were similar in between the open field and enriched box (*P* = 0.808, Figure 1A, lower-left) during the 30 min exposure duration.

On the other hand, mice showed significant higher levels defecation (*P*<0.001) and escape behaviors (*P*<0.05) in the open field compared with the enriched box (Figure 1A, lower panels). In addition, the amount of defecation in the enriched box (1.83±0.42) were not significantly different from that in the homecage (0.90±0.35; *P* = 0.10); the escape behaviors were also rarely seen in either the enriched box or the homecage (Figure 1A and Figure 4A). Together, these results demonstrate that under our experimental conditions, anxiety was elicited in the open field (but not in the enriched box) and was irrelevant to locomotor activity.

### Responsiveness of BLA neurons in the open-field test

A total of 180 well isolated units were recorded from eight mice subjected to open-field and enriched box tests. Of these, 38 neurons (21%) exhibited one of three firing patterns upon exposing to these two environments: (1) activated only in the open field; (2) activated in both environments; (3) suppressed in both environments.

The first group of BLA neurons (type-1; n = 10) showed a distinct tonic firing pattern in the open field box: their firing rates increased gradually, reaching the maximal values within 15–20 min, and then maintained at high levels during the rest of the open-field tests (Figure 1B, left). Importantly, this group of BLA neurons did not show significant change in firing rate in the enriched box (Figure 1B, right). These discharge properties correlated well with the animals' anxiety-like behaviors that were only evoked in the open field but not the enriched box (Figure 1A, lower panels), suggesting that the activity of this group of BLA neurons was likely to represent anxiety or emotional values of anxiety triggered by the open-field test.

To further investigate the dynamic activity of these anxiety-related BLA neurons, the mean firing rates in each 5-min time window were calculated during the entire period of open-field tests for all recorded type-1 BLA neurons. The average firing rates of these neurons increased slowly and gradually in the open field, but not in the enriched box (Figure 1C, left). We therefore wanted to know whether the anxiety level and anxiety-like behaviors increase slowly in a similar manner. Thus anxiety-like behaviors were measured in both the open field and the enriched boxes during the first 10 min and the last 20 min of the testing periods, respectively. The levels of the anxiety-like behaviors were not different in the first 10 min, but were significantly different in the last 20 min between the open field and the enriched box tests (Figure 1C, right), indicating that anxiety-like behaviors also develop slowly and gradually in the open field. Overall, these type-1 BLA neurons increased their firing rates gradually in parallel to a gradual development of anxiety-like behaviors under conditions of anxiety. We therefore suggest that these BLA neurons responsed selectivily for anxiety in the open field test.

**Figure 2:**
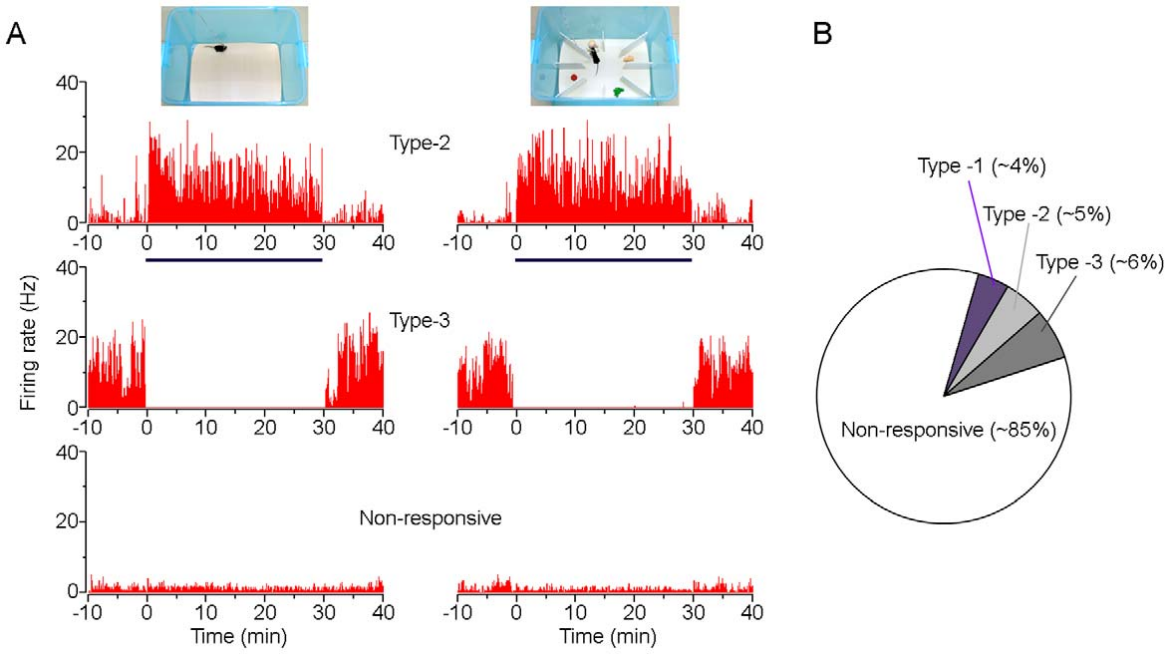
BLA neurons differentially respond to novel environmental exposure. (A) Rate histograms of three simultaneously recorded BLA neurons (type-2, top panels; type-3, middle panels; and a non-responsive neuron, bottom panels) during the open field (left) and enriched box (right) exposure. (B) Percentages of the each type of BLA neurons.

The second group of BLA neurons (type-2; n = 13) increased their firing rates (Figure 2A, top panel) and the third group (type-3; n = 15) decreased their firing rates (Figure 2A, middle panel) in both the open field and the enriched box tests. These non-selectivity response properties did not correlate with animals' anxiety-like behaviors (that were selectively evoked in the open field; Figure 1A), suggesting that they were unlikely to encode anxiety. They may be involved in encoding uncertainty or saliency caused by environmental changes.

### Responsiveness of type-1 BLA neurons in elevated plus-maze test

**Figure 3:**
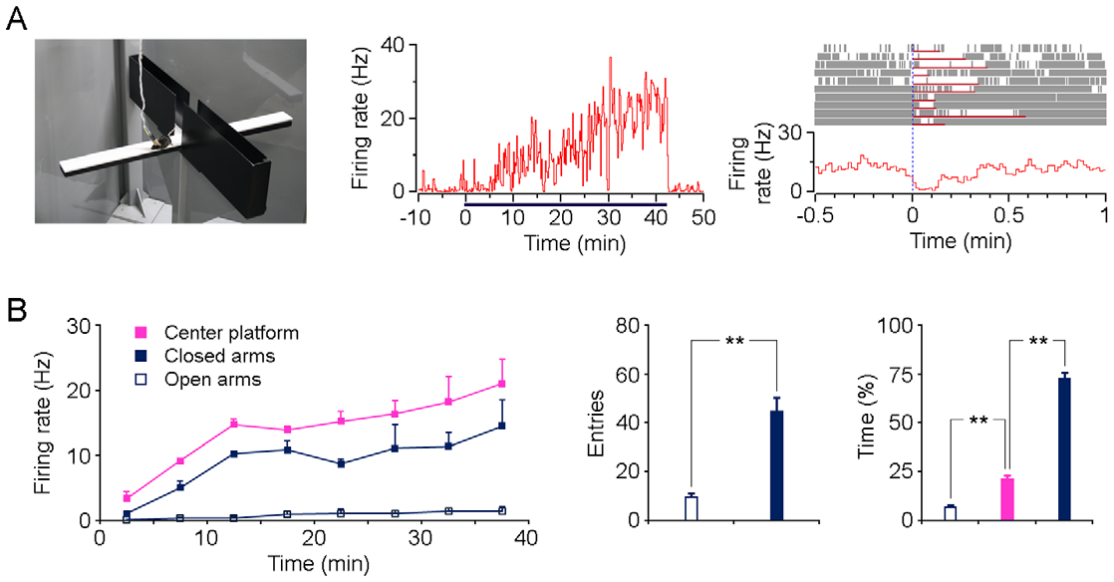
Activity of type-1 BLA neurons in the elevated plus-maze test. (A) Left panel, EPM test. Middle panel, rate histogram of one type-1 BLA neuron during the EPM test. Horizontal line below the histogram indicates the duration of EPM exposure. Right panel, perievent raster plot (10 trials) and perievent histogram of the same neuron referenced to the entries into the open arms. Red horizontal lines below each raster plot indicate the durations of each open-arm exploration. (B) Left panel, average firing rates of type-1 BLA neurons on open arms, closed arms and the center platform during the EPM exposure (n = 4; firing rates were sampled and averaged every 5 min). Right panels, mice made significantly fewer entries into the open arms than into the closed arms (left), and spent significantly less time in open arms (right). Data in all panels are plotted as mean ± SEM (error bars). **P<0.002 by *t*-test.

The elevated plus-maze test is also commonly used to examine anxiety in rodents [24], [25]. Prolonged exposure of the rodent to an EPM causes anxiety and stimulates the expression of the immediate-early gene *c-Fos* in the amygdala [26]. To determine whether type-1 BLA neurons were also involved in processing anxiety in EPM, activities of BLA neurons were recorded in freely-behaving mice exposed to an EPM (Figure 3A, left). Firing rates of the type-1 BLA neurons increased gradually during the EPM test (Figure 3A, middle), in a similar manner to their responses to the open-field test. Surprisely, they were almost silent whenever the mice explored the unprotected area of the open arm (Figure 3A, right). We speculated this silence of these neurons was probably due to the introduction of fear stimuli (such as height). Collectively, type-1 BLA neurons increased slowly on both the closed arms and the center platform; in contrast, they displayed very limited or no activity on the open arms (Figure 3B, left). Notably, the activity of these neurons was higher on the center-platform than on the closed arms (*P*<0.01, two-way analysis of variance). Behaviorally, mice entered the closed arms more frequently and spent most of the time on the closed arms (Figure 3B, middle and right).

### Responsiveness of type-1 BLA neurons to open-field-like environments

**Figure 4:**
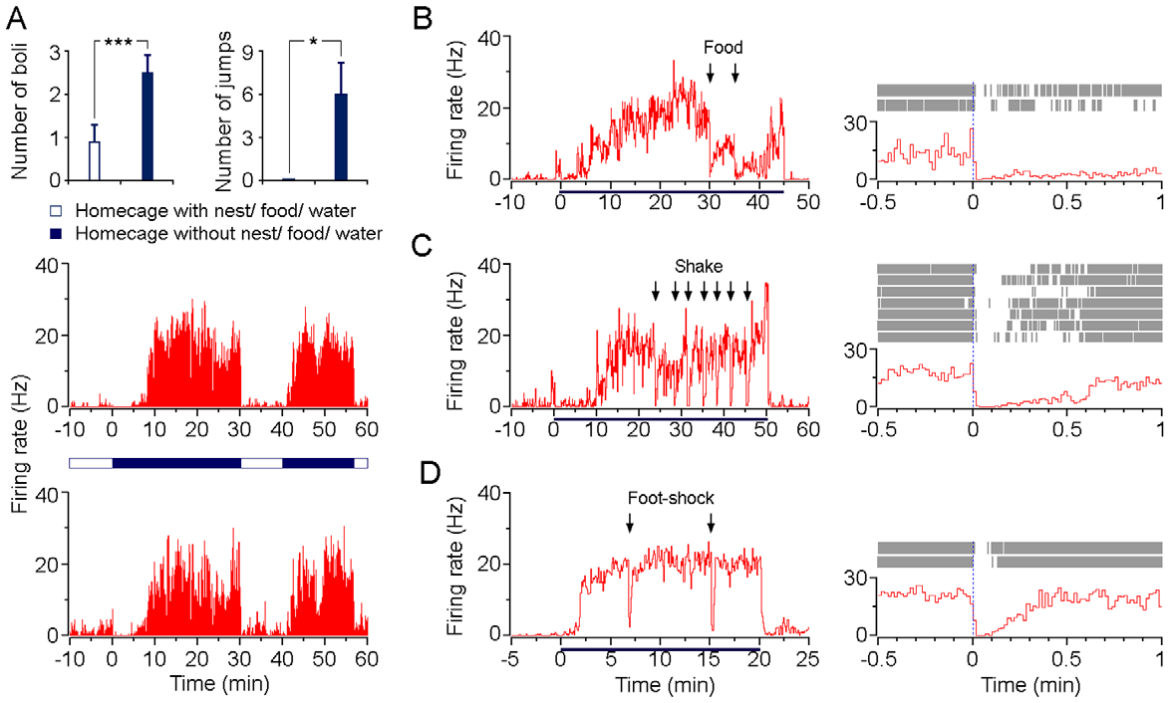
Responsiveness of type-1 BLA neurons to environmental changes. (A) Upper panels, anxiety-like behaviors were significantly elicited when nest/food/water cup were removed from homecage (n = 10; mean ± SEM). *P<0.05, ***P<0.001 by *t*-test. Lower panels, rate histograms of two simultaneously recorded type-1 BLA neurons in response to the removal (twice; at 0:00 & 40:00, respectively) and return (twice; at 30:00 & 56:30, respectively) of the nest/food/water cup in the homecage. (B-D) Left panels, rate histograms of type-1 BLA neurons in response to open-field-like environmental exposures and external stimuli deliveries. Horizontal lines below each histogram indicate the exposure durations. Arrows indicate the time onset of food delivery (B), shake stimuli (C) and foot-shocks (D). Right panels, perievent raster plots and the perievent histograms referenced to each external stimulus on the left, respectively.

To further examine the association between anxiety and these type-1 BLA neurons, we carried out another set of experiment. We took away the nest (with cotton), the water cup and the rodent diets from the homecage to generate an open-field-like environment. The removal of these items evoked anxiety-like behaviors in mice (Figure 4A, upper), while simultaneously type-1 BLA neurons showed significant activation (Figure 4A, lower). The firing pattern of these neurons was similar to their responses to the open-field test: a slow-onset and a steady increase in firing rates. Moreover, the activity of these neurons were rapidly and strongly suppressed after the nest, food and water cup were returned to the homecage (Figure 4A, lower). These results further support the notion that the activity of these type-1 BLA neurons represents anxiety and/or emotional values of anxiety.

Previous studies suggest that anxiety levels decline during attentional distractions [27],[28]. We also found that the activity of type-1 BLA neurons was suppressed during the exploration of the open arms in EPM tests (Figure 3A, right). To determine whether the tonic firing patterns of type-1 BLA neurons can be blocked by additional novel stimuli, mice were placed in the open-field-like environments to induce anxiety, then were stimulated with either comfortable (e.g. food) or uncomfortable (e.g. shaking and foot-shocks) stimuli. Food strongly suppressed the activity of type-1 BLA neurons (Figure 4B). Aversive stimuli, such as shaking and foot-shocks, also potently inhibited their activity (Figure 4, C and D). These results suggest that anxiety might be mitigated by natural approaches (such as introducing novel stimuli) at least in part due to the inhibition of these type-1 BLA neurons.

### Activation of type-1 BLA neurons correlates with levels of anxiety

**Figure 5:**
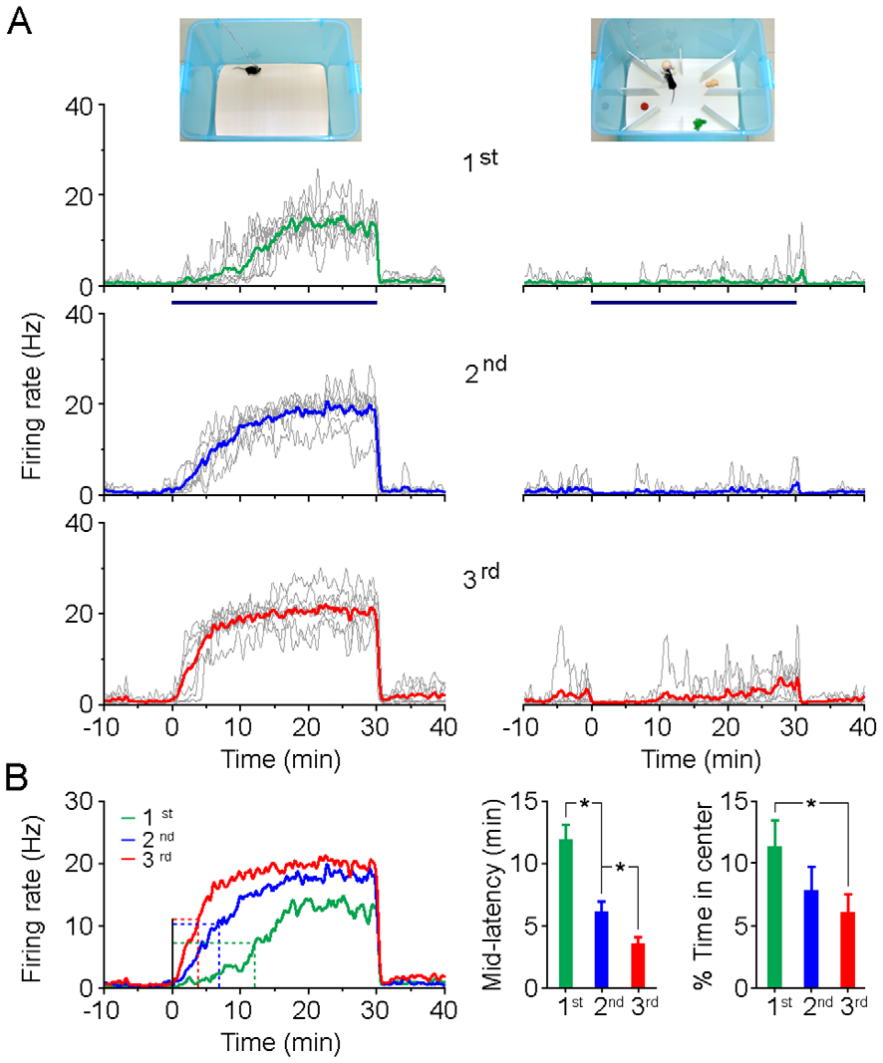
Activation of type-1 BLA neurons correlates with levels of anxiety. (A) Rate histograms of type-1 BLA neurons (n = 7) during repeated exposure to an open field (left panels) and an enriched box (right panels). Green, blue and red lines represent average responses while grey lines for each individual neuron. Horizontal lines below the histograms indicate the exposure duration (30 min). (B) Left panel, average rate histograms show increased activity of type-1 BLA neurons in repeated open field tests. Horizontal dotted line marked the half maximum firing rates of type-1 neurons; vertical dotted line marked the latencies of half maximum firing rates. Middle and right panels, mid-latencies of maximum firing rate of type-1 neurons (n = 7; mean ± SEM) and time spent in the center of the open field (n = 10; mean ± SEM) in repeated open-field tests. *P<0.05 by *t*-test.

It has been shown that repeated open-field tests increased the levels of anxiety-related behaviors [29]. In agreement with these observations, we found that mice showed decreased exploration of the center area (an index to estimate anxiety) during repeated open-field tests over three successive trials (Figure 5B, right). We therefore set to examine whether the type-1 BLA neurons also showed enhanced activity during repeated open-field tests. The average firing rate histogram clearly demonstrated that the onset activity of these anxiety-related neurons was progressively enhanced in each succeeding trial (Figure 5 A and B, left panels). Analysis of the latencies of the median value of the maximum firing rates showed that there were significant differences for each comparison (Figure 5B, middle panel; *P*<0.05). In contrast, the same type-1 BLA neurons showed very limited activity during repeated exposure to the enriched environment (Figure 5A, right panels). These results further suggest that activation of these type-1 BLA neurons correlates with the levels of anxiety states in an open field environment. Additionally, these observations also raise an intriguing possibility that repeated exposure to an open field may elicit synaptic plasticity in the BLA neural circuitry, thus resulting in increased excitability of these BLA anxiety-related neurons.

## Discussion

BLA is widely believed to play an important role in fear and anxiety [3], [30], [31]. Although much evidence implicates the role of the amygdala in anxiety [6], [7], little data is available regarding the neuronal correlates of anxiety in the amygdala. In this study, we have identified one group of BLA neurons (type-1) that are possibly involved in coding anxiety and/or emotional values of anxiety. First, these BLA neurons were activated in multiple animal models of anxiety (e.g., open field test, elevated plus maze test). Moreover, all impoverished open-field-like environments tested (including impoverished homecage; Figure 4A) in our experiment evoked strong activation of these type-1 BLA neurons. This increased activity cannot be simply explained by response specificity or selectivity of the environmental stimuli (e.g., novelty, familiarity) [32], [33].

Second, different from fear, anxiety is a characteristically slower-onset, longer-lasting emotional process, and evoked under potential threats even in the absence of direct danger [34]. In consistent with this notion, our data showed that the anxiety-related activity of these BLA neurons increased gradually and then maintained high activity in both the open-field and elevated plus-maze tests. The characteristic late-onset and tonic firing patterns of BLA anxiety-related neurons correlated well with the slow-onset anxiety-like behaviors in the absence of direct danger. Moreover, aversive or fearful stimuli (e.g., foot-shock) strongly suppressed, rather than increasing the activity of these neurons, suggesting that these BLA neurons were more likely to process or encode anxiety rather than fear.

It is also interesting to note that the activity of these BLA anxiety-related neurons was suppressed by introduced external stimuli, which was independent of the stimulus properties (e.g., comfortable or uncomfortable). Previous studies have demonstrated that the anxiety level declines when an outside stimulus is introduced or the animal's attention is shifted away [27], [28]. Our findings further support the notion that anxiety state might be suppressed or shifted at least temporarily by introduction of external stimuli.

More importantly, results reported here revealed that excitability of BLA anxiety-related neurons increased progressively during repeated exposures to anxiety conditions; in parallel, the levels of anxiety also increased gradually (Figure 5). These results raised an important question regarding whether abnormally elevated activation of these BLA anxiety-related neurons might be a key component of the neural mechanisms that mediate anxiety disorders. Additionally, anxiety is likely to elicit synaptic plasticity in BLA [35], [36], thus increasing the strength of synaptic transmission and excitability of BLA anxiety-related neurons. Therefore, these BLA anxiety-related neurons are likely to serve as a potential therapeutic target in treating excessive anxiety and anxiety disorders.

## Methods and Materials

### Subjects

All mouse work described in this study have been conducted according to Animals Act, 2006 (China) and approved by the Institutional Animal Care and Use Committee (IACUC approval ID #M07015) of the East China Normal University. Male C57BL/6J mice (3–8 months old) were singly housed in the customized homecages (55 cm in diameter, 42 cm in height; with nest/food/water) and kept on a 12 hr light/dark schedule. Data from eight mice from which we stably recorded multiple neuronal activities in the BLA were used in the current analyses (Figure S1 and S2). Another 12 mice were used for behavioral tests.

### Microdrive Construction and Surgery

We designed a 64-channel (a bundle of 16 tetrodes), ultra-light (<2 g) and movable recording microdrive (Figure S1) similar to that described previously [37]. This design enables adjustable advancement of the implanted electrode bundle when turning a small screw nut on the microdrive. Tetrodes were constructed by twisting together four 13-µm nichrome wires (Stablohm 675, California Fine Wire, CA, USA) and melting their insulation; wire tips were gold-plated to reduce the impedance to 0.5–1 MΩ at 1 kHz before surgery. Mice were frequently handled before surgery. On the day of surgery, a mouse was anesthetized with i.p. injection of pentobarbital sodium (∼40 mg/kg), and the body temperature was kept constant by a thermoregulatory (FHC Inc., Bowdoin, ME, USA). Then subcutaneous layer of tissue was removed to expose the skull. The position for electrode implantation (1.7 mm posterior to bregma, 3.1 mm laterally) was measured and marked, and then a hole at this position was drilled on the skull. A microdrive was positioned and the 16 tetrodes array was lowered through the drilled hole into above the amygdala (3.5 mm ventral from brain surface). The gaps between the electrodes and hole were filled with softened paraffin and the microdrive was secured with dental cement.

### In Vivo Recording

Methods were similar to that described previously [37]. In brief, spikes (filtered at 400-7k Hz; digitized at 40 kHz) were recorded during the whole experimental process using the Plexon multichannel acquisition processor system (Plexon Inc., Dallas, TX, USA); units were isolated using the Plexon OfflineSorter (Figure S2). The final recording position was marked by passing a 10-sec 20-µA current through two selected electrodes. Mice were then perfused with 0.9% saline followed by 4% paraformaldehyde. Brains were then cut and stained with cresyl violet (Figure S1 C and D).

### Behavioral Tests

#### Open field test

A mouse was picked up and placed in the center of the open field arena (55×40×40 cm; illuminated to 50 lux) and allowed to explore the open field freely for 30 min. The following parameters were counted: distance moved (measured by Plexon CinePlex system); percentage of time spent in a center zone (25×20 cm) of the open field; number of fecal boli; and number of jumps (all four paws in the air followed with a landing sound). The same open field test was repeated three times with a 24-hour-interval between the first and the second trials and a 3-hour-interval between the second and the third. For enriched box (55×40×40 cm; divided into eight small rooms and enriched with toys) test, the same procedures and measurements as the open field test were followed.

#### Elevated Plus Maze (EPM) test

The EPM was a plus-shaped maze composed of a central platform (5×5 cm) and two open and two closed arms (35×5 cm each). The closed arms are enclosed by 15 cm-high walls. The maze was elevated 40 cm from base and illuminated to 50 lux. During a typical test, a mouse was placed on the central platform facing an open arm and allowed to explore the maze for 40 minutes. Each test was recorded by the Plexon CinePlex system and scored by the observer. Times spent on open arms, closed arms and central platform, entries into open and closed arms were scored. An arm entry was scored when the mouse placed four paws onto the arm.

#### Food exposure

Mouse was placed in a novel open field (55 cm in diameter, 42 cm in height). After about 30 min free exploration, mouse favored food (e.g. sugar, rice, chocolate) was placed in the center of the open field by the experimenter, and mice were allowed to free exploration or consummation of the food.

#### Shake and foot-shock events

The shake box was a square box (15×15×18 cm) made of plastic boards and fixed on a vortex machine. After about 20 min free exploration in the shake box, seven randomized shake stimuli (3 sec) were delivered to the mouse unexpectedly through a computer controlled mechanic interface, with an interval of 3∼5 min between trials. The foot-shock box was a square box (20×20×30 cm) with a 16-bar inescapable shock grid floor (H10-11M-TC; Coulbourn Instruments, Whitehall, PA, USA). After an 5–10 min free exploration in the shock box, two or three scrambled foot shock (0.5 mA, 1 sec) were delivered to the mouse unexpectedly, with an interval of 5∼10 min.

### Data Analysis

Anxiety-like behaviors, elevated plus maze explorative behaviors were scored by the experimenter. Results were expressed as Mean ± SEM. Statistical significances were calculated using two-tailed paired t-test unless otherwise noted. Sorted spikes were analyzed using NeuroExplorer (Nex Technologies, USA) and Matlab (Mathworks, USA). Rate histograms, peri-event rasters and histograms were presented as single neuronal responses unless otherwise noted.

## Supporting Information

Figure S1 **Multi-channel** ***in vivo*** **recording from the basolateral amygdala in freely behaving mice.** (A) A 64-channel (16-tetrode), movable (screw-driven) microdrive array. (B) A freely behaving mouse implanted with the 64-channel microdrive array. (C) A coronal Nissl-stained section indicating the recording position in the BLA. (D) Coronal diagrams showing locations of the electrode tips in the BLA across 8 mice. Numbers to the right, Anteroposterior coordinates (in millimeters) caudal to bregma.
(TIF)

Figure S2 **Stable recording and isolation of BLA neurons.** (A) Three well-isolated units (units 1-3) recorded from one tetrode clustered in *Plexon Offline Sorter* (top panel) and the representative waveforms for each unit on day 1 (bottom panels). (B and C) The same three units (as shown in A) and their representative waveforms on days 7 and 14.
(TIF)
