# Supplementary figures and images for: Neurons in the Amygdala with Response-Selectivity for Anxiety in Two Ethologically Based Tests
Source: PLoS One. 2011 Apr 11;6(4):e18739. doi: 10.1371/journal.pone.0018739 (PMC3073991; doi:10.1371/journal.pone.0018739)

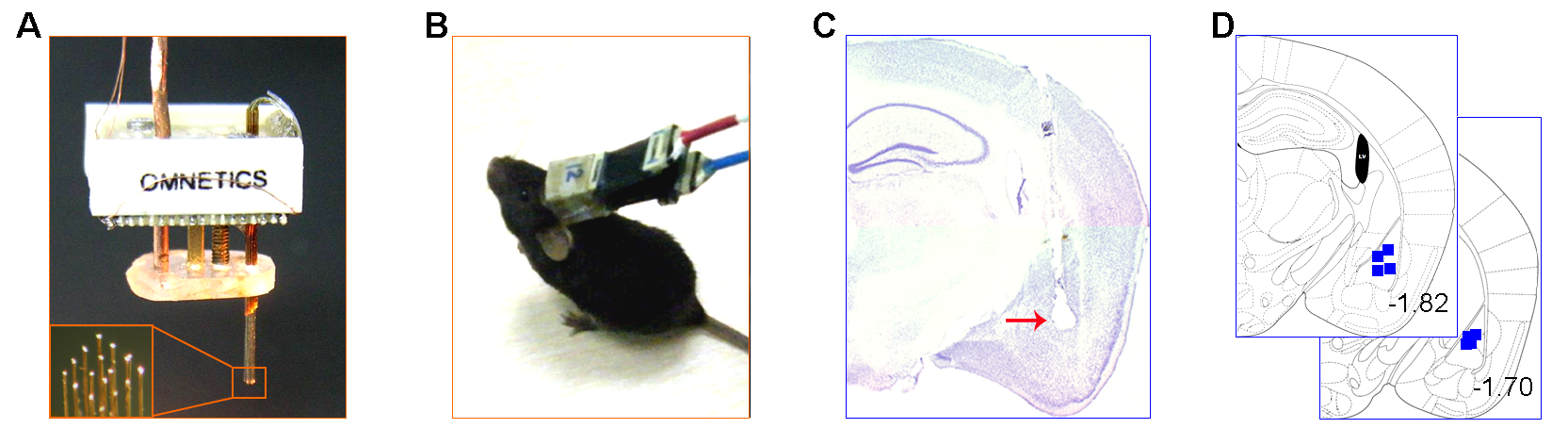

Supplement: Figure S1 — Multi-channel in vivo recording from the basolateral amygdala in freely behaving mice. (A) A 64-channel (16-tetrode), movable (screw-driven) microdrive array. (B) A freely behaving mouse implanted with the 64-channel microdrive array. (C) A coronal Nissl-stained section indicating the recording position in the BLA. (D) Coronal diagrams showing locations of the electrode tips in the BLA across 8 mice. Numbers to the right, Anteroposterior coordinates (in millimeters) caudal to bregma. (TIF) [file pone.0018739.s001.tif]

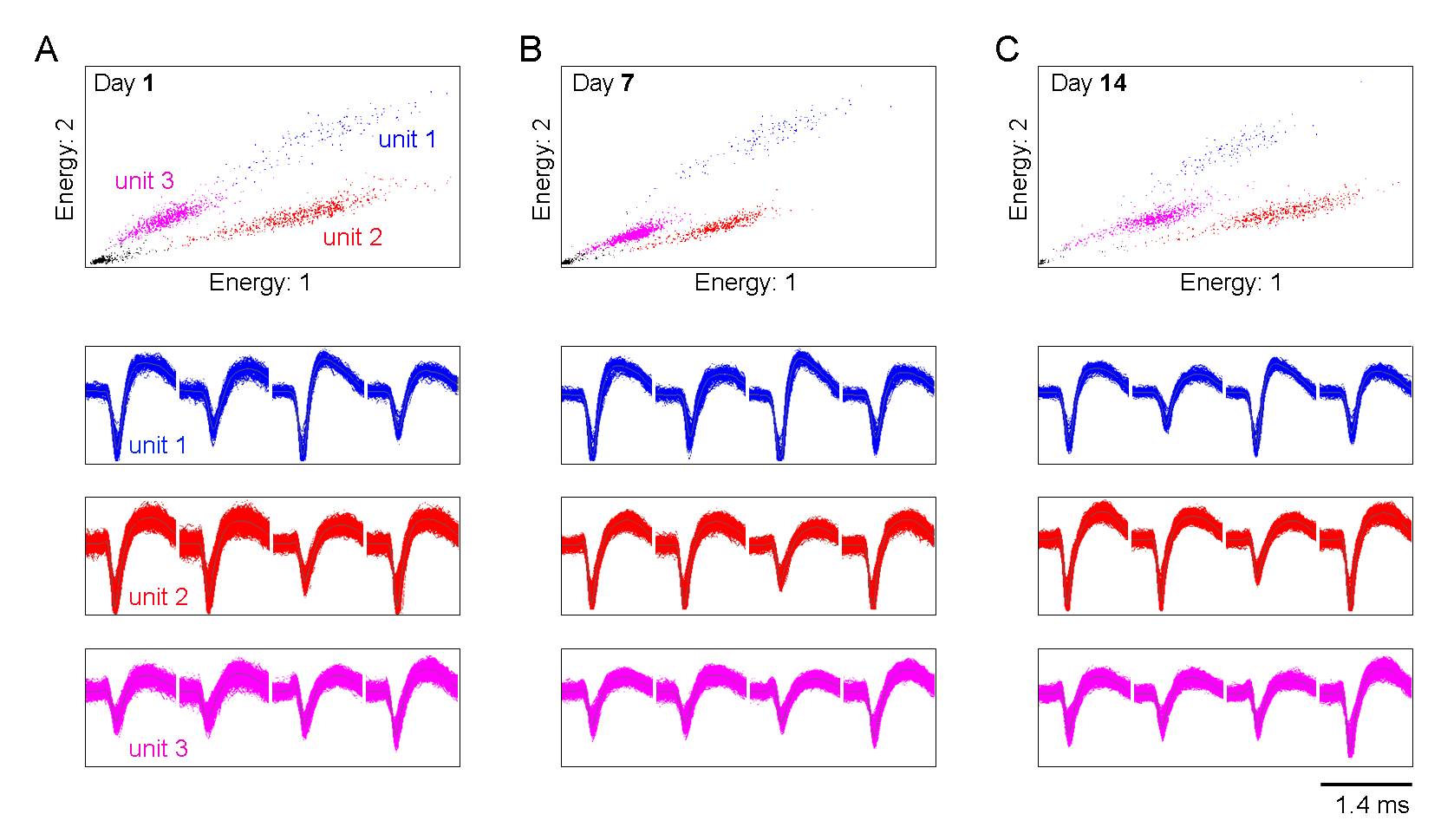

Supplement: Figure S2 — Stable recording and isolation of BLA neurons. (A) Three well-isolated units (units 1-3) recorded from one tetrode clustered in Plexon Offline Sorter (top panel) and the representative waveforms for each unit on day 1 (bottom panels). (B and C) The same three units (as shown in A) and their representative waveforms on days 7 and 14. (TIF) [file pone.0018739.s002.tif]
